# Impact of trimester-specific nutrition counseling on maternal vitamin D status, and perinatal outcomes in India: a quasi-experimental cohort study

**DOI:** 10.1186/s12889-026-27315-2

**Published:** 2026-05-08

**Authors:** Mohd Faisal Khan, Mohammed Al-Zharani, Ashok Khurana, K. Aparna Sharma, Aruna Nigam, Sana Alam, Fahd A. Nasr, Wajhul Qamar, Md Ghaznavi Idris, Shakilur Rahman, Kapil Dev

**Affiliations:** 1 Department of Biotechnology, Jamia Millia Islamia, New Delhi, 110025 India; 2 Department of Biology, College of Science, Imam Mohammad Ibn Saud Islamic University (IMSIU), Riyadh, 11623 Saudi Arabia; 3 Department of Fetal Medicine, The Ultrasound Lab, New Delhi, 110024 India; 4 Department of Obstetrics and Gynaecology, All India Institute of Medical Sciences, New Delhi, 110029 India; 5 Department of Obstetrics and Gynaecology, Hamdard Institute of Medical Sciences and Research, New Delhi, 110062 India; 6 Department of Biochemistry, Hamdard Institute of Medical Sciences and Research, New Delhi, 110062 India; 7 Department of Pharmacology and Toxicology, College of Pharmacy, King Saud University, Riyadh, 11451 Saudi Arabia; 8 Department of Bioengineering and Technology, Gauhati University, Guwahati, Assam 781014 India

**Keywords:** Nutritional counseling, Maternal nutrition, Vitamin D deficiency, Public health, Pregnancy outcomes

## Abstract

**Background:**

Vitamin D deficiency affects more than 90% of Indian pregnant women, yet its isolated impact on perinatal outcomes remains unclear amid multifactorial nutritional challenges. These pose a challenge to achieving SDG 3.2 targets for reducing neonatal mortality. This study evaluated the effects of trimester-specific nutritional counseling on maternal vitamin D status, nutritional practices, and perinatal outcomes in an Indian cohort.​

**Methods:**

A quasi-experimental prospective cohort study was conducted among 173 (88 intervention, 85 control) pregnant women (8–14 weeks of gestation) at a tertiary hospital in New Delhi between October 2023 and May 2025. The intervention included individualized counseling on vitamin D-rich foods, protein, lipids, and weight management at 8–14, 24–28, and 32–36 weeks, along with supplementation. Controls received standard care. Outcomes included serum 25(OH)D, nutrient intake, gestational weight gain, birth weight, and mode of delivery. Analyses used t-tests, correlations, and multivariate regression (*p* < 0.05, FDR-adjusted).​

**Results:**

A total of 173 participants completed the study. Baseline severe vitamin D deficiency improved significantly more in the intervention group than in the control group, with a large effect size (Cohen’s d = 0.97). Protein intake increased, and nutrition knowledge scores improved substantially following the intervention (*p* < 0.05). Birth weights were comparable between groups, and no significant association was observed between maternal vitamin D levels and birth weight. Instead, gestational weight gain, protein intake, and caloric intake emerged as significant predictors of low birth weight (OR 0.73–0.995), while vitamin D was not a significant factor. Higher maternal BMI was associated with an increased likelihood of cesarean delivery (OR 0.87, *p* < 0.05).

**Conclusion:**

Trimester-specific nutritional counseling improved maternal vitamin D status and dietary practices but was not significantly associated with birthweight. Overall nutritional adequacy, particularly protein intake and gestational weight gain, appeared more closely related to fetal growth. These findings should be interpreted in light of the study design and warrant confirmation in larger studies.

**Supplementary Information:**

The online version contains supplementary material available at 10.1186/s12889-026-27315-2.

## Introduction

Pregnancy is a critical period of physiological transformation that necessitates significant metabolic and nutritional adjustments to support fetal development and maternal well-being. Among the micronutrients essential for a healthy pregnancy, vitamin D plays a complex role in cellular differentiation, immune system regulation, and placental and fetal growth. Vitamin D deficiency has become a global epidemic, affecting 1 billion people worldwide despite sunlight availability, particularly in low- and middle-income countries (LMICs) such as India, where the prevalence among pregnant women can reach up to 93.5%, even in areas with ample sunlight, creating a “silent emergency” that undermines maternal-fetal health [1].

The paradox of widespread vitamin D deficiency in sun-rich countries like India stems from multiple intersecting factors, including limited sun exposure due to indoor lifestyles and cultural clothing practices, along with biological determinants such as darker skin pigmentation, which reduces dermal vitamin D synthesis, and low dietary intake of fortified or vitamin D-rich foods [2, 3]. These environmental and behavioral factors often intersect with sociodemographic inequalities, increasing maternal nutritional vulnerabilities. Deficiency during pregnancy is increasingly seen as a risk factor for adverse maternal and neonatal outcomes, including preeclampsia, gestational diabetes mellitus (GDM), fetal growth restriction, preterm birth, low birth weight (LBW), small for gestational age (SGA), and cesarean birth [4, 5]. These challenges directly threaten SDG 3.2 targets for reducing neonatal mortality to ≤ 12/1000 live births by 2030, particularly in India, where low birth weight accounts for 40% of under-5 deaths [6, 7].

India’s maternal health scene is further complicated by a dual burden of malnutrition, where undernutrition and increasing overweight/obesity coexist among women of reproductive age [8]. This epidemiological shift, driven by rapid urbanization, dietary shifts toward energy-dense processed foods, and sedentary lifestyles, results in a diverse maternal body mass index (BMI) profile. Both low and high maternal BMI have been associated with poor pregnancy outcomes, partly through altered micronutrient metabolism, including vitamin D bioavailability [9]. Obesity, in particular, may lower circulating 25(OH)D levels due to sequestration in fat tissue, thereby worsening the deficiency and related risks [10].

Globally, studies from China, Norway, and Greece have confirmed links between low maternal vitamin D levels and complications such as preeclampsia and fetal growth issues [3, 9, 11]. Similar evidence from African and European populations shows that inadequate or excessive weight gain during pregnancy, often related to BMI, contributes to low birthweight, macrosomia, and delivery problems [12, 13]. However, in the Indian context, where unique cultural dietary habits, limited access to nutrient-rich foods, and deep-rooted health inequalities significantly influence pregnancy nutrition, there remains a significant evidence gap. Specifically, few studies have systematically examined the interactions among maternal BMI, vitamin D levels, and perinatal outcomes, or assessed the impact of food-based prenatal counseling on addressing these complex issues.

International research has highlighted the potential of trimester-specific nutritional interventions, particularly in promoting healthy dietary habits and improving micronutrient intake. For instance, studies from Southeast Asia have demonstrated that structured nutritional counseling can significantly increase vitamin D levels and improve pregnancy outcomes [14]. However, evidence from India remains limited, particularly regarding the actual impact of counseling-based interventions integrated into routine antenatal care. Additionally, supplementation alone may not be practical or sustainable in many resource-limited settings, underscoring the need for behavioral and food-based strategies for long-term health improvements. Therefore, this study aimed to assess maternal nutritional status and nutrient intake during pregnancy, with a specific focus on vitamin D levels and their relationship with maternal BMI and perinatal outcomes.

## Materials and methods

### Study design

This quasi-experimental prospective cohort study with a pre-post intervention design was conducted at the Department of Obstetrics and Gynecology of a tertiary hospital in New Delhi between October 2023 and May 2025. The site was selected for its socioeconomic diversity to ensure a representative sample. Ethical approval was obtained from the relevant authority (*HIMSR/IEC/00149/2023*,* dated 4 October 2023)*, and written informed consent was obtained from all participants prior to participation.

### Sample size and study samples

**Fig. 1:**
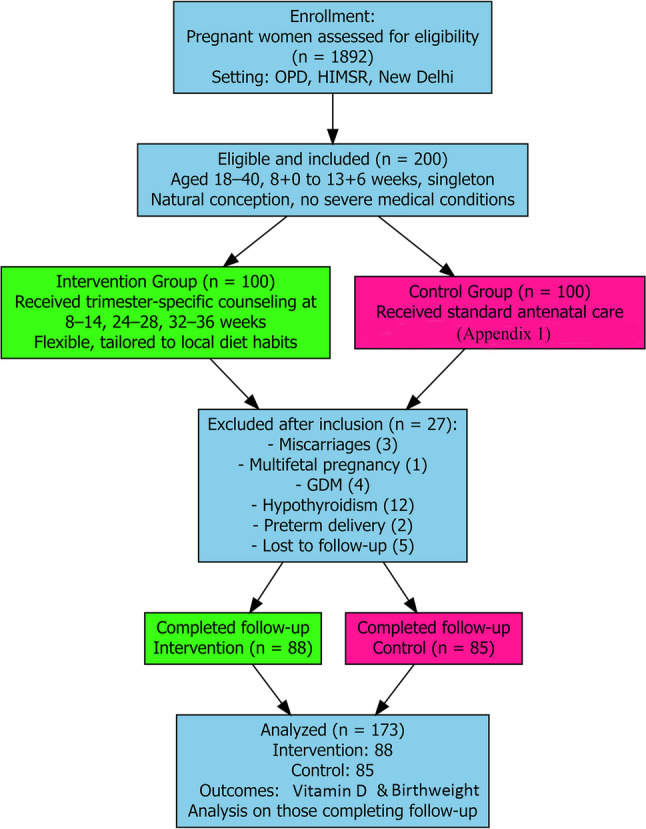
Flowchart outlining the number of participants screened, excluded, allocated into groups, and who completed the study in the intervention and control groups

The sample size was calculated based on estimates from previous studies, assuming a 95% confidence level, 5% margin of error, and a 20% anticipated dropout rate; the final sample size was 200 (adjusted from 192). A total of 1892 pregnant women were screened among outpatient department (OPD) attendees for eligibility. Of these, 200 met the inclusion and exclusion criteria and were enrolled, constituting the baseline sample. Participants were allocated to intervention (*n* = 100) and control (*n* = 100) groups, forming the baseline sample. Following enrolment and baseline assessments, 27 participants were excluded from the final analysis due to the unavailability of follow-up data, either because follow-up samples could not be obtained or participants were lost to follow-up, as detailed in Fig. 1. The final analytical sample comprised 173 participants (intervention *n* = 88; control *n* = 85). Participants were assigned consecutively (non-randomly) to intervention groups based on clinic attendance patterns during defined enrolment periods. A sequential allocation sampling (rather than randomization) was used due to logistical constraints that prevented simultaneous randomization in this public hospital setting. This study included pregnant women aged 18–40 years, who were 8 + 0 to 13 + 6 weeks of gestation, with spontaneous singleton pregnancies and no major pre-existing medical conditions. Those outside the age/gestational range, with multifetal pregnancies, ART conception, or significant comorbidities (including the incidence of GDM during pregnancy) were excluded.

### Intervention & follow-up

The intervention group received trimester-specific, individualized nutrition counseling for 15–20 min at 8–14, 24–28, and 32–36 weeks of gestation. A trained dietitian delivered all sessions. The counseling content was based on ICMR RDA and adapted to local cultural dietary patterns. Counseling was modified when women reported dietary restrictions, such as vegetarian diets or cultural limitations. Alternative nutrient sources were suggested to maintain adequacy. The sessions emphasized general pregnancy-related nutrition, including balanced meals, macronutrient distribution, and key micronutrient supplementation (IFA/vitamin D), as well as strategies to manage gestational weight gain and metabolic parameters, using pictorial handouts. The intent was to reinforce healthy eating throughout pregnancy. Personalization was guided by each participant’s dietary habits, BMI category, weight trajectory, and relevant antenatal findings such as hemoglobin or lipid levels. The control group received routine prenatal care. Follow-up assessments were conducted at baseline and again in the third trimester. Counseling was tailored to each woman’s needs in the intervention group across the successive trimesters. IOM weight-gain guidelines were used as a reference (*Appendix I (S1)*).

### Data collection tools and procedures

Baseline and post-intervention data were collected using a pre-designed questionnaire on socio-demographics, obstetric history, anthropometry, dietary intake, and nutrition knowledge, administered through a structured one-to-one interviewer-administered questionnaire (24-hour dietary recall of two non-consecutive days and a food frequency questionnaire). The nutrition knowledge questionnaire was developed based on existing literature and adapted to the local context [15–19] (*Appendix II (S2)*). It was pilot tested with 20 participants to assess clarity, comprehension, and feasibility. Content validity was further evaluated by an expert panel comprising three clinicians and two registered dietitians, who reviewed the questionnaire for relevance, clarity, and cultural appropriateness. Based on their feedback, minor modifications were made to improve item clarity. However, formal external validation in an independent population was not performed. Religious affiliation was collected because it is closely linked to culturally specific nutritional practices in the study setting, for example, vegetarianism, avoidance of beef or pork, and fasting customs, which can directly influence nutrient intake during pregnancy. BMI was calculated from self-reported pre-pregnancy weight, and gestational weight gain was monitored against the IOM guidelines once per trimester using calibrated digital scales. Fetal biometric data, such as abdominal circumference (AC) and estimated fetal weight (EFW), were obtained from second- and third-trimester ultrasound reports. Delivery outcomes, including mode of delivery and neonatal birth weight, were recorded from hospital registers and verified against antenatal records.

Biochemical assessments included serum vitamin D levels, measured at baseline and in the third trimester using an Abbott Architect i2000SR analyser, with inter- and intra-assay variability kept below 2%. A level of < 20 ng/mL was considered vitamin D deficient. Maternal nutrition knowledge was assessed pre- and post-intervention using a structured questionnaire developed from existing literature, pilot tested for clarity and feasibility, and reviewed by an expert panel for content validity, covering awareness, food literacy, and dietary practices.

### Statistical analysis

Data were analyzed using SPSS (IBM SPSS Statistics 27.0.1.0), RStudio (version 4.4.2), and Microsoft Excel (Microsoft 365). Data distribution was assessed using the Shapiro-Wilk test and visual inspection of Q-Q plots. Given the approximate normal distribution and the sample size, parametric tests were considered appropriate, supported by the Central Limit Theorem.

Primary analyses were pre-specified based on the study objectives and included paired and independent t-tests to assess within- and between-group differences, as well as Pearson correlation and multivariate regression models to examine associations between maternal nutritional factors, including vitamin D, and perinatal outcomes. Multivariate linear and logistic regression analyses were used to identify predictors of maternal vitamin D levels, birthweight, and mode of delivery. Additional analyses, including extended correlation assessments and LASSO regression, were conducted as exploratory to further investigate potential relationships. The Benjamini-Hochberg method was applied to control the false discovery rate. Statistical significance was set at *p* < 0.05.

## Results

A total of 173 participants completed the study (intervention *n* = 88; control *n* = 85), with losses due to miscarriage [3], GDM [4], hypothyroidism [12], preterm delivery [2], loss to follow-up [5], and one with a multifetal pregnancy, shown in the flowchart (Fig. 1).

### Maternal anthropometry and gestational weight gain

Baseline characteristics were comparable between the intervention and control groups, including age, anthropometry, gestational age, gravidity distribution, religion, dietary habits, education levels, socioeconomic status, and blood pressure. No notable differences were observed across groups in any key demographic or clinical parameters, and none were significant (*p* > 0.05) (*Supplementary Table; ST1*). The mean maternal age was 27.23 ± 4.25 years in the intervention group and 27.59 ± 4.23 years in the control group. Mean weight (56.78 ± 11.89 kg vs. 54.07 ± 9.42 kg) and height were similar (154.89 ± 5.01 cm vs. 154.61 ± 6.48 cm, respectively). This balanced distribution of baseline characteristics supports comparability between the study groups and reduces potential confounding in the analysis.

Pre-pregnancy BMI distribution showed that 14.5% (*n* = 25) of participants were underweight and 31.2% (*n* = 54) were overweight or obese. Both groups showed a steady increase in gestational weight and BMI throughout the trimesters, with changes in Δ weight (kg) and Δ BMI (kg/m²) across the trimesters. These changes consistently reflected a progressive increase in weight and BMI, as summarized in Table 1.

**Table 1:** Trimester-wise changes in Maternal Anthropometry, Nutrient Intake, Knowledge scores, and Vitamin D Status from Baseline to Third Trimester. Values are reported as mean ± SD. Δ indicates change from baseline to follow-up

Parameter	Intervention (*n* = 88)	Control (*n* = 85)
Anthropometric Measures		
Pre-pregnancy BMI (kg/m²)	23.57 ± 4.37	22.61 ± 3.57
1st Trimester BMI (kg/m²)	24.28 ± 4.24	23.26 ± 3.93
3rd Trimester BMI (kg/m²)	27.24 ± 4.14	26.46 ± 3.93
Δ Weight (kg)		
Weight gain: Pre-pregnancy → 3rd trimester)	8.97 ± 3.64	8.53 ± 4.27
Weight gain: Pre-pregnancy → 1st Trimester (kg)	1.75 ± 2.60	1.54 ± 2.30
Weight gain: 1st → 2nd Trimester (kg)	3.85 ± 2.10	4.04 ± 2.18
Weight gain: 2nd → 3rd Trimester (kg)	3.26 ± 1.76	3.61 ± 1.86
Δ BMI (kg/m²)		
BMI gain: Pre-pregnancy → 3rd trimester)	3.67 ± 1.44	3.86 ± 1.83
BMI gain: Pre-pregnancy→ 1st Trimester (kg/m²)	0.71 ± 1.08	0.66 ± 1.01
BMI gain: 1st → 2nd Trimester (kg/m²)	1.62 ± 0.91	1.74 ± 0.94
BMI gain: 2nd → 3rd Trimester (kg/m²)	1.34 ± 0.70	1.50 ± 0.75
Dietary Intake		
Total Energy Intake (kcal/day): Baseline	1405.29 ± 194.22	1395.37 ± 153.20
Total Energy Intake: Follow-up	1510.05 ± 191.48	1562.8 ± 171.63
Protein Intake (g/day): Baseline	50.20 ± 9.48	49.28 ± 9.48
Protein Intake (g/day): Follow-up	57.54 ± 9.51	49.85 ± 7.34
Δ Protein Intake (g/day)	+ 7.34	+ 0.57
Fat Intake (g/day): Baseline	48.77 ± 9.41	54.91 ± 10.62
Fat Intake (g/day): Follow-up	58.44 ± 11.41	61.51 ± 11.92
Vitamin D Level		
Baseline Vitamin D (ng/mL)	12.04 ± 10.82	12.72 ± 6.78
Follow-up Vitamin D (ng/mL)	37.33 ± 24.44	22.73 ± 11.80
Δ Vitamin D (ng/mL)	+ 25.29	+ 10.01
Took Vitamin D Supplements (%)	69% (61/88)	28.23% (24/85)
Knowledge Score		
Nutrition Knowledge Score (Baseline)	5.58 ± 3.09	5.63 ± 2.93
Nutrition Knowledge Score (Post)	10.30 ± 1.94	5.87 ± 2.75

Paired t-tests confirmed significant trimester-wise increases in BMI (*p* < 0.05) and weight (*p* < 0.05) within the intervention group. In contrast, the control group demonstrated smaller, non-significant changes in these measures (*p* > 0.05). Gestational weight gain was positively correlated with caloric intake in the intervention group (*r* = 0.231, *p* < 0.05).

### Nutrient intake, nutritional knowledge & practices, and supplementation

Nutrient intake improved across pregnancy in both groups, as summarized in Table 1. Protein intake increased from 50.20 ± 9.48 g/day to 57.54 ± 9.51 g/day in the intervention group (*p* < 0.05); minimal change was seen in the control group (49.28 ± 9.48 g to 49.85 ± 7.34 g/day). The intake of fruits and green leafy vegetables improved more in the intervention group, whereas the intake of vitamin D-related dietary sources, such as milk, eggs, meat, and fish, improved in both groups during the study period.

Nutrition knowledge improved in the intervention group (baseline: 5.58 ± 3.09; post: 10.30 ± 1.94; *p* < 0.05). Iron-folic acid supplementation adherence reached 100%, and vitamin D supplementation increased to 69% in the intervention group (*p* < 0.05) from 0% at baseline. In contrast, the control group showed no significant changes in knowledge (5.63 ± 2.93 to 5.87 ± 2.75; *p* > 0.05) but a slight shift in supplement adherence (vitamin D: 0% at baseline to 28.23% at follow-up). No supplementation was prescribed to the control group; however, approximately 28% of participants reported taking vitamin D supplements independently, likely based on prior prescriptions, personal health practices, or previous pregnancy experience.

### Serum vitamin D levels

**Fig. 2:**
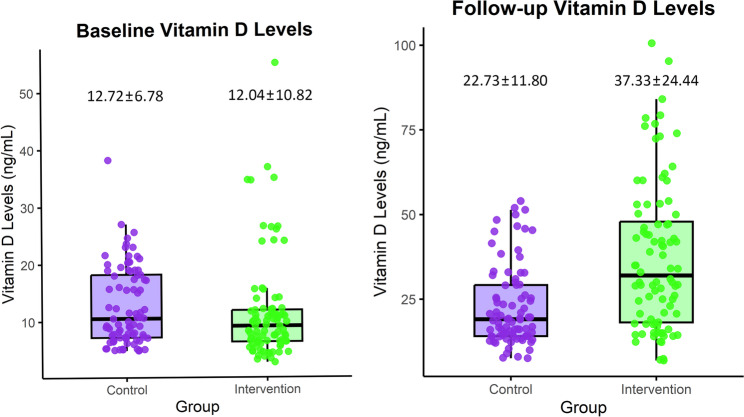
Scatter boxplot illustrating baseline and follow-up serum vitamin D levels (ng/mL), in the intervention and control groups

Baseline Vitamin D levels indicated severe deficiency in both groups: intervention group (12.04 ± 10.82 ng/mL) and control group (12.72 ± 6.78 ng/mL). At follow-up, Vitamin D concentrations improved significantly in both groups (*p* < 0.05), with the intervention group achieving higher concentrations (37.33 ± 24.44 ng/mL) compared to the control group (22.73 ± 11.80 ng/mL; *p* < 0.05) (Fig. 2). The between-group difference at follow-up was 14.60 ng/mL (95% CI: 20.10 to 8.63), reflecting a large effect size (Cohen’s d = 0.75).

Within-group improvements in serum vitamin D levels were also statistically significant in both groups. In the intervention group, the mean difference from baseline was 25.29 ng/mL (95% CI: 30.79 to 19.79; Cohen’s d = 0.97), whereas in the control group, the mean improvement was 10.01 ng/mL (95% CI: 12.66 to 7.36; Cohen’s d = 0.81). These within-group effect sizes indicate a larger magnitude of improvement in the intervention group. These results remained statistically significant after correction for false discovery rate (FDR).

### Correlates and predictors of vitamin D levels

**Fig. 3:**
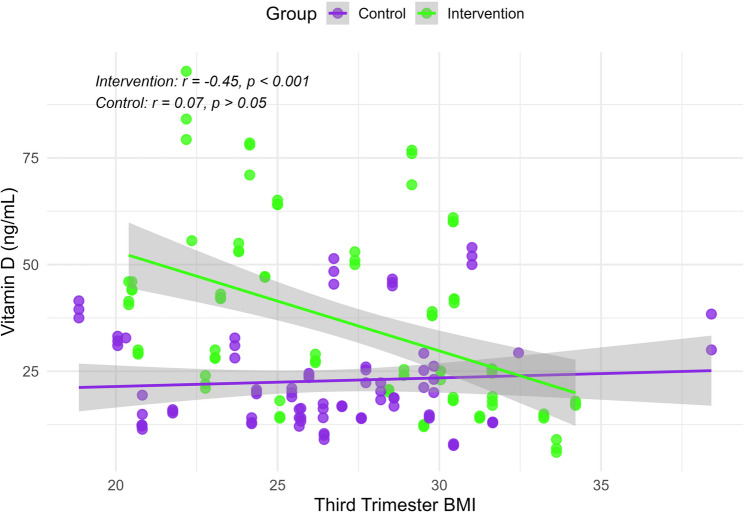
Scatter plot with fitted regression lines illustrating the correlation between third-trimester BMI and follow-up serum vitamin D levels in intervention and control groups

Vitamin D levels were inversely correlated with third-trimester BMI and weight at follow-up in the intervention group (BMI: *r* = -0.452, *p* < 0.05; weight: *r* = -0.514, *p* < 0.05). No significant correlations were observed between third-trimester BMI and weight, and vitamin D levels in the control group (*p* > 0.05) (Fig. 3).

Multivariate linear regression in the intervention group showed that third-trimester BMI, age, protein intake, and carbohydrate intake were significant negative predictors of vitamin D levels (all *p*-values < 0.05). In contrast, caloric intake and consumption of eggs, meat, and fish were significant positive predictors (all *p*-values < 0.05). The model explained 46.8% of the variance (*R*² = 0.468, *p* < 0.05). In the control group, vitamin D was positively associated with caloric intake and fat intake while nut and seed intake showed a negative association (all *p*-values < 0.05). The control model explained 29.1% of the variance (*R*² = 0.291, *p* < 0.05). Statistically significant results remained consistent after applying the FDR correction in both groups.

### Fetal biometry, birthweight, and vitamin D

Fetal biometry showed expected growth throughout gestation, with abdominal circumference and estimated fetal weight strongly associated with birth weight. Maternal vitamin D levels were not significantly associated with fetal growth parameters. Neonatal outcomes were comparable between groups (Table 2).

**Table 2:** Fetal Biometry, Perinatal Outcomes, and Associations with Maternal Vitamin D and BMI. Values are reported as the mean ± SD and as percentages

Parameter	Intervention (*n* = 88)	Control (*n* = 85)
Gestational Age at Delivery (weeks)	38.26 ± 1.19	38.80 ± 1.40
Birthweight (g)	3,049.4 ± 434.9	3,000.4 ± 469.2
Low Birthweight (< 2,500 g)	12.5%	16.5%
Preterm Births (%)	6.81%	4.71%
APGAR Score at 1 min	8.00 ± 0.69	7.95 ± 0.43
APGAR Score at 5 min	8.90 ± 0.55	8.85 ± 0.45
Mode of Delivery: Vaginal (%)	59.1%	65.88%
Mode of Delivery: Cesarean (%)	40.9%	34.12%
Vitamin D: Vaginal Delivery (ng/mL)	40.33 ± 25.43	23.85 ± 11.66
Vitamin D: Cesarean Delivery (ng/mL)	33.01 ± 22.57	20.56 ± 11.96
Vitamin D: Term Births (ng/mL)	38.39 ± 24.51	22.90 ± 11.96
Vitamin D: Preterm Births (ng/mL)	15.15 ± 1.97	19.25 ± 8.11
Maternal Hemoglobin at Birth (g/dL)	11.7 ± 0.96	10.60 ± 1.33
Maternal Calcium at Birth (mg/dL)	9.02 ± 0.20	8.89 ± 0.42
Fetal Biometry-2nd Trimester		
Abdominal Circumference (cm)	14.49 ± 1.40	14.89 ± 1.93
Estimated Fetal Weight (g)	319.53 ± 77.70	347.05 ± 105.94
Fetal Biometry-3rd Trimester		
Abdominal Circumference (cm)	28.30 ± 1.36	28.84 ± 2.13
Estimated Fetal Weight (g)	2012.11 ± 248.60	2069.67 ± 366.45
Correlations (r, *p*-value)		
AC vs. EFW (2nd trimester)	*r* = 0.910, *p* < 0.05	*r* = 0.910, *p* < 0.05
AC vs. EFW (3rd trimester)	*r* = 0.866, *p* < 0.05	*r* = 0.866, *p* < 0.05
2nd Trimester BMI vs. 3rd Trimester AC	*r* = -0.281, *p* < 0.05	*p* > 0.05
2nd Trimester BMI vs. 3rd Trimester EFW	*r* = -0.367, *p* < 0.05	*p* > 0.05
3rd Trimester BMI vs. 3rd Trimester AC	*r* = -0.268, *p* < 0.05	*p* > 0.05
3rd Trimester BMI vs. 3rd Trimester EFW	*r* = -0.341, *p* < 0.05	*p* > 0.05
3rd Trimester TG vs. EFW	*r* = 0.244, *p* < 0.05	*p* > 0.05
Neonatal BW vs. 3rd Trimester AC	*r* = 0.567, *p* < 0.05	*r* = 0.267, *p* < 0.05
Neonatal BW vs. 3rd Trimester EFW	*r* = 0.665, *p* < 0.05	*r* = 0.241, *p* < 0.05

**Fig. 4:**
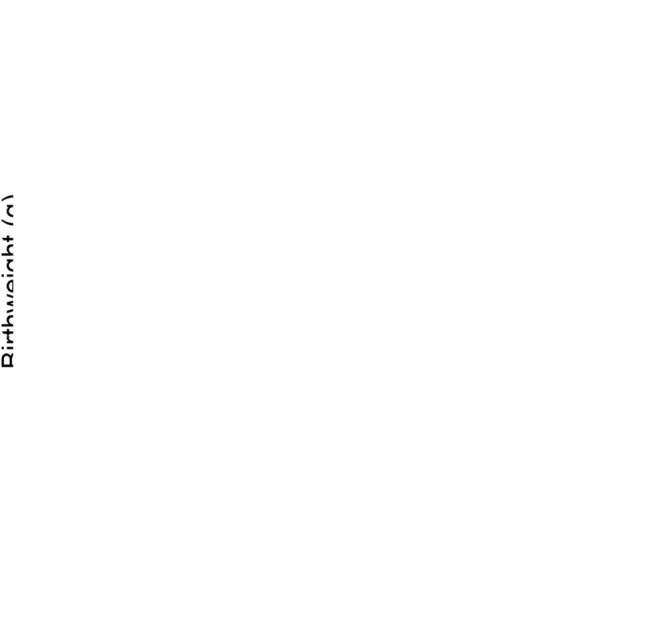
Scatter plot with fitted regression lines showing the association between follow-up serum vitamin D levels and neonatal birthweight in intervention and control groups, with fitted regression lines and 95% confidence intervals

Follow-up vitamin D levels were not significantly associated with birthweight in either group (intervention: *r* = 0.148, *p* > 0.05; control: *r* = 0.168, *p* > 0.05) (Fig. 4), and no differences were observed across birthweight categories. These findings remained consistent after multiple comparison adjustment.

Analysis of follow-up vitamin D levels by birth weight category showed that the mean vitamin D level was 33.8 ± 21.2 ng/mL in the low birth weight (LBW) group (*n* = 24), 29.7 ± 20.4 ng/mL in the normal birth weight group (*n* = 148), and 7.6 ng/mL in the macrosomia group (*n* = 1). One-way ANOVA across birthweight categories revealed no significant group differences in vitamin D levels (F = 1.011, *p* > 0.05; *η*² = 0.01). Results remained non-significant after FDR adjustment.

### Predictors of low birthweight

Multivariate logistic regression identified gestational weight gain (OR = 0.82, 95% CI: 0.69–0.96, *p* < 0.05), protein intake (OR = 0.73, 95% CI: 0.58–0.89, *p* < 0.05), and caloric intake (OR = 0.995, 95% CI: 0.991–0.998, *p* < 0.05) as significant protective factors against LBW. Follow-up vitamin D levels (OR = 1.02, 95% CI: 1.00-1.05, *p* > 0.05) and third-trimester BMI (OR = 1.14, 95% CI: 0.97–1.35, *p* > 0.05) were not statistically significant predictors. All reported *p*-values for LBW predictors were adjusted using the Benjamini-Hochberg method, with protective associations for calorie intake, protein intake, and gestational weight gain remaining statistically significant. The model demonstrated acceptable discrimination (AUC ≈ 0.78), although calibration was suboptimal (Hosmer-Lemeshow *p* < 0.05), indicating limited model fit and potentially affecting the precision of the estimated associations. LASSO regression retained gestational weight gain, dietary factors, BMI, vitamin D, and knowledge score as key predictors.

### Mode of delivery

Higher third-trimester BMI was associated with cesarean delivery. In the intervention group, the mean BMI for cesarean deliveries was 28.2 vs. 26.6 for vaginal deliveries (*p* < 0.05). In the control group, the association was significant (*p* < 0.05), with an OR of 0.87 (95% CI: 0.76–0.98, *p* < 0.05). Vitamin D levels did not significantly differ between cesarean and vaginal deliveries in either the total sample or within groups (*p* > 0.05). Logistic regression analysis revealed that follow-up vitamin D levels were not a significant predictor of mode of delivery (OR = 1.01, 95% CI: 0.99–1.03, *p* > 0.05), and the results remained unchanged after multiple-comparison correction. LASSO regression confirmed third-trimester BMI as a predictor of cesarean delivery, while vitamin D and parity were not retained.

## Discussion

This study demonstrates that a structured nutritional intervention can significantly improve maternal nutritional knowledge, dietary practices, and serum vitamin D concentrations among pregnant women in India.

The high prevalence of severe vitamin D deficiency at baseline in our cohort underscores a critical public health concern among pregnant women in India. Our results are consistent with findings from across the Indian subcontinent and other parts of Asia, where limited sun exposure and dietary habits contribute to widespread insufficiency among pregnant women, exceeding the prevalence typically reported in many European and North American populations [20–22]. While our intervention successfully raised the mean vitamin D level in the intervention group to a sufficient level (37.33 ng/mL), a significant proportion of the control group remained insufficient (22.73 ng/mL), highlighting the inadequacy of routine care alone in addressing this widespread deficiency. The magnitude of improvement in our intervention group is comparable to that observed in controlled trials in Europe and North America, which have similarly demonstrated the efficacy of targeted supplementation and dietary counseling in correcting maternal vitamin D status in other at-risk populations [23].

A central finding of this study is the dissociation between improved maternal vitamin D status and its impact on neonatal birth weight. Despite the large effect size of the intervention on serum vitamin D, we observed no significant correlation between maternal vitamin D levels and birth weight, nor a significant reduction in the incidence of low birth weight (LBW) in the intervention group. This finding contributes to an ongoing and often conflicting body of evidence in the literature. While some observational studies and meta-analyses, particularly in Caucasian populations, have suggested a link between low maternal vitamin D and adverse perinatal outcomes, including LBW and preterm birth, results from randomized controlled trials have been inconsistent [24–26]. A large trial in the United Kingdom and Bangladesh, as well as a recent meta-analysis of studies from diverse global settings, including data from African and Asian populations, reported, similar to our results, that vitamin D supplementation during pregnancy did not significantly reduce the risk of LBW [27]. This contrasts with findings from a European cohort, which reported a positive association between maternal vitamin D and birth weight, suggesting that the impact may be context-dependent and potentially modulated by factors such as baseline vitamin D status, ethnicity, and coexisting nutritional deficiencies [28]. Our results suggest that in a population where multiple nutritional factors are at play, correcting a single micronutrient deficiency, even a severe one, may be insufficient to overcome the multifactorial determinants of fetal growth.

Instead, our analysis identified adequate gestational weight gain, along with sufficient protein and caloric intake, as powerful and independent predictors of birthweight and as protective factors against LBW. This reinforces the fundamental principle of maternal-fetal medicine: fetal growth depends on the mother’s overall nutritional state and energy balance. This finding is consistent with global evidence from Asia, sub-Saharan Africa, and high-income European and American cohorts, which consistently identifies insufficient gestational weight gain and poor protein-energy intake as leading preventable causes of fetal growth restriction and LBW [28–30]. Our findings support SDGs 3.1 and 3.2 by identifying scalable protein-energy interventions that may contribute to reductions in low LBW cases in India, thereby helping meet global targets for maternal (≤ 70/100,000) and neonatal mortality [31, 32]. The strong protective effect of protein intake observed in our study is particularly noteworthy and aligns with evidence emphasizing its crucial role in the development of placental and fetal tissue [33]. This finding suggests that public health strategies to reduce LBW in India should prioritize ensuring adequate maternal protein and calorie intake, as well as micronutrient supplementation.

The inverse correlation observed between third-trimester BMI and vitamin D levels in the intervention group corroborates established physiological mechanisms, including the volumetric dilution and sequestration of vitamin D in adipose tissue, a phenomenon documented across diverse populations [34, 35]. Furthermore, our findings reaffirm the strong association between higher maternal BMI and an increased likelihood of cesarean delivery, a well-established risk factor in global obstetric practice [36]. Large cohort studies in the United States, Europe, and China have consistently reported that maternal obesity is a strong independent risk factor for both emergency and elective cesarean sections, mediated through factors such as labour dystocia and fetal macrosomia [37, 38]. Our results confirm that this association holds true in an Indian population, adding to the evidence that managing gestational weight gain is crucial not only for fetal outcomes but also for mitigating obstetric complications.

The intervention’s success in improving dietary quality without leading to excessive gestational weight gain is a positive secondary outcome, suggesting that such programs can promote healthier pregnancies without increasing the risk of weight-related complications. This may reflect broader lifestyle shifts in engaged participants. However, the absence of an association between maternal vitamin D levels and mode of delivery suggests that the clinical implications of correcting vitamin D deficiency may be more specific to maternal health than to broader obstetric outcomes.

Overall, the findings emphasize the importance of a more integrated approach to maternal nutrition during pregnancy. While vitamin D status improved substantially following the intervention, it was not associated with birthweight in this cohort. In contrast, gestational weight gain and overall dietary intake, particularly protein and caloric adequacy, showed more consistent associations with fetal growth. These observations may be relevant to ongoing public health programs such as POSHAN 2.0 and Anaemia Mukt Bharat, which could benefit from incorporating a more comprehensive nutritional framework that addresses both macro- and micronutrient needs. The observed association between higher maternal BMI and cesarean delivery further supports the inclusion of maternal weight monitoring in routine antenatal care.

However, this study has several limitations. The quasi-experimental design and non-random allocation may introduce selection bias and limit causal inference, and residual confounding cannot be excluded. Although the sample size was adequate for primary analyses, it may limit the stability of multivariable models and increase the risk of overfitting. In addition, the combined use of dietary counseling and supplementation precludes complete separation of their independent effects. The nutrition knowledge questionnaire, although pilot-tested and reviewed for content validity, was not externally validated, which may affect its generalizability and measurement precision.

In summary, markers of overall nutritional adequacy, including protein intake and gestational weight gain, were more consistently associated with fetal growth than maternal vitamin D status. The interpretation of these findings is constrained by the study design and sample size, and further large-scale, randomized studies are needed to better define the relative contributions of micronutrient status and overall dietary intake to perinatal outcomes.

## Conclusion

Trimester-specific nutritional counseling improved maternal vitamin D status, dietary practices, and nutritional knowledge; however, no significant association was observed between maternal vitamin D status and birth weight. In contrast, gestational weight gain and overall dietary intake emerged as more consistent determinants of fetal growth in this cohort. These findings suggest that broader nutritional adequacy may be more relevant to perinatal outcomes than isolated micronutrient correction, while acknowledging the need for confirmation in large-scale, randomized studies to better delineate the relative contributions of micronutrients and overall dietary intake to perinatal outcomes.

## Supplementary Information

Supplementary Material 1.

## Data Availability

On request after 2027, as the data is part of a Ph.D. thesis.

## Abbreviations

AC: Abdominal Circumference
APGAR: Activity, Pulse, Grimace, Appearance, Respiration
ART: Assisted Reproductive Technology
AUC: Area Under Curve
BMI: Body Mass Index
CI: Confidence Interval
EFW: Estimated Fetal Weight
FDR: False Discovery Rate
GDM: Gestational Diabetes Mellitus
GWG: Gestational Weight Gain
IFA: Iron-Folic Acid
ICMR: Indian Council of Medical Research
IOM: Institute of Medicine
LBW: Low Birth Weight
LMICs: Low- and Middle-Income Countries
LASSO: Least Absolute Shrinkage and Selection Operator
OPD: Outpatient Department
OR: Odds Ratio
POSHAN: Prime Minister’s Overarching Scheme for Holistic Nourishment
R²: Coefficient of Determination
RCH: Reproductive and Child Health
RDA: Recommended Dietary Allowance
SD: Standard Deviation
SDG: Sustainable Development Goal
SGA: Small for Gestational Age
SPSS: Statistical Package for the Social Sciences
ST: Supplementary Table
TG: Triglycerides
25OHD: 25-Hydroxyvitamin D
