# Supplementary material for: Impact of trimester-specific nutrition counseling on maternal vitamin D status, and perinatal outcomes in India: a quasi-experimental cohort study
Source: BMC Public Health. 2026 May 8;26:1558. doi: 10.1186/s12889-026-27315-2 (PMC13173945; doi:10.1186/s12889-026-27315-2)
Supplement: Supplementary file 1 — Supplementary Material 1. [file 12889_2026_27315_MOESM1_ESM.pdf]

Supplementary Material (S1)

Routine Antenatal Care (for Control Group)

Initial Visit (8-14 Weeks)

- *Medical History and Physical Exam:* Assess maternal health, including BMI, blood pressure, and medical history (e.g., diabetes, hypertension). Identify socio-cultural or economic barriers (e.g., access to nutrient-rich foods).
- *Screening Tests:* Blood tests for hemoglobin, blood group, Rh factor, and infections (e.g., HIV, syphilis). Urine tests for protein and glucose.
- *Ultrasound:* Confirm gestational age and detect multiple pregnancies or anomalies, NT-NB scan.
- *Interventions:* Initiate iron-folic acid to prevent neural tube defects and calcium

Second Trimester (18-28 Weeks)

- *Monitoring:* Check blood pressure, weight gain, and fetal growth. Anomaly scan and screen for gestational diabetes (oral glucose tolerance test (OGTT) at 24-28 weeks).
- *Supplementation:* Continue iron and calcium.
- *Tetanus Vaccination:* Administer tetanus toxoid (two doses, one month apart).

Third Trimester (32-40 Weeks)

- *Monitoring:* Regular checks for preeclampsia (blood pressure, urine protein), fetal position, and growth. Screen for anemia and adjust iron supplementation.
- *Preparation for Delivery:* Discuss birth plans, breastfeeding, and postpartum care.

Nutrition Education Sessions during Pregnancy (for Intervention Group)

In addition to routine antenatal care, the intervention group received structured and individualized nutrition education, trimester-specific nutrition counseling delivered in person by a qualified dietitian (*each session of individualized nutrition counseling lasted for 15-20 minutes)*, provided at three pivotal gestational stages: baseline (8-14 weeks), midpoint (24-28 weeks), and post-intervention (32-36 weeks). Each session focused on personalized meal planning tailored to individual needs, utilizing evidence-based recommendations that incorporated 24-hour dietary recall, food frequency data, BMI category, and accounting for diverse nutritional practices (e.g., vegetarianism prevalent in Hinduism, where many people avoid animal proteins like fish, meat, or eggs, and some exclude roots, tubers, garlic, or onions, posing challenges to nutrient intake). Supplementation was tailored to meet individual physiological needs and health requirements. The content was adapted from the ICMR and WHO dietary guidelines for pregnant women. The counseling emphasized:

BASELINE SESSION (8-14 Weeks)

- The baseline session established the foundation for healthy dietary practices in early pregnancy.
- Counseling focused on the importance of consuming a balanced diet incorporating complex carbohydrates like whole grains and millets, high biological value lean proteins (for non-vegetarian) and legumes and beans (for vegetarian or vegan), and healthy fats such as nuts and seeds while advising against ultra or highly processed foods, excessive caffeine, and unsafe items like raw eggs or unpasteurized milk products.
- Participants were also informed and educated on the RDA set by ICMR-National Institute of Nutrition, including additional calorie intake (no significant increase in calories is typically needed in the first trimester, as per RDA) in each trimester, protein intake, and the amount of micronutrient intake.
- Underweight patients were counselled to increase energy and protein intake from complex carbohydrates and good sources of fat. For protein intake, participants were asked to obtain protein from lean sources, such as chicken and fish, in the case of non-vegetarians. Vegetarians were given the options of various lentils and paneer (cottage cheese).

- Overweight patients were counselled to eat a balanced diet. They were asked to consume nutrient-rich foods, such as lean protein and complex carbohydrates, and avoid calorie-dense foods like fried food items, packaged snacks, and bakery goods.
- Strategies to address common early pregnancy issues, such as nausea and loss of appetite, were addressed with practical suggestions like consuming small, frequent meals, ginger-based remedies to tackle nausea, and maintaining hydration.
- The importance of micronutrients, especially iron, calcium, folic acid, and vitamin D, was emphasized. Along with food sources, participants learned about foods that enhance iron absorption (e.g., vitamin C) and those that inhibit it (e.g., tannin-rich tea and coffee).
- A weekly dose of vitamin D supplementation of 60K (Cholecalciferol 60,000 IU) was prescribed for 8-10 weeks to expectant mothers who were vitamin D deficient (<20ng/mL), depending on the severity.
- Micronutrient supplementation was a key component; emphasis was put on taking and continuing with folic acid, iron supplementation, and calcium supplementation from the 15th week of gestation.
- They were told not to take iron and calcium supplements together. Additionally, avoid taking iron with calcium-rich foods, such as milk or dairy products, and instead take it with vitamin C for improved absorption.
- Participants were also informed and educated about how maternal lipid profiles and hemoglobin levels influence fetal outcomes, introducing the relevance of lipid and anemia management early in pregnancy.
- A printed handout summarizing dos and don'ts was provided *(Source of Handout~ attached at the end: Dietary guidelines for Indians from ICMR-National Institute of Nutrition).*

MID-POINT SESSION (24-28 Weeks)

- This session addressed the trimester-specific increased nutritional demands of the second trimester (as per RDA: additional 350 Kcal and 9.5g Protein per day), focusing on glycemic and lipid regulation, alongside personalized/individualized weight management.

- Participants were educated and encouraged to consume low-glycemic index foods (e.g., legumes, green vegetables), omega-3-rich foods (e.g., flaxseed, walnuts, fish for non-vegetarians), and avoid sugar-laden snacks and unsaturated fats.
- Participants were also counseled on label reading, salt and fat intake reduction, and avoidance of ultra-processed foods.
- The importance of vitamin D, both through dietary sources and limited sun exposure, was also highlighted.
- Dietary advice was customized for participants with inadequate or excessive weight gain, ensuring balanced energy intake without compromising nutrient quality.
- Underweight participants were guided to include energy-dense foods like nut butters and milk-based smoothies; overweight participants received portion control strategies to balance calories without nutrient loss, ensuring nutrient sufficiency, and to control calorie-dense foods.
- Personalized meal planning guidance and portion guides were given to help implement dietary modifications based on the RDA laid by the ICMR.
- For those at risk of gestational diabetes or lipid imbalances, receive tailored strategies with nutrient-rich foods to optimize metabolic health.

POST-INTERVENTION SESSION (32-36 Weeks)

- The final session focused on preparing for childbirth and sustaining nutritional improvements.
- This session also addressed the trimester-specific increased nutritional demands of the third trimester (as per RDA: additional 450 Kcal and 22g Protein per day), focusing on glycemic and lipid regulation, alongside personalized/individualized weight management.
- Weight management strategies were reviewed to support optimal fetal growth and reduce complications such as anemia, macrosomia, or preterm birth.
- Nutritional counseling emphasized iron-rich, nutrient-dense foods and bioavailability-enhancing combinations to address late-pregnancy issues like constipation.
- High-fiber foods, calcium-rich choices, and hydration were recommended for constipation, fatigue, and cramps.
- Participants were encouraged to continue healthy habits postpartum.

Note: *All supplements were prescribed by doctors after reviewing lab reports (e.g., vitamin D deficiency) and discussion with the dietitian to ensure safety and personalization.*

Summary

**Session**	**Gestational Age**	**Key Focus Areas**	**Strategies and Tools Used**
**Early Pregnancy**	8–14 weeks	Balanced diet, symptom relief, micronutrient needs such as iron, calcium, folic acid, vitamin D, lipids, Hb, and lipid/anemia awareness	Handouts and one-to-one interactive counseling
**Mid-Pregnancy**	24–28 weeks	Blood sugar/lipid control, weight management, and vitamin D focus	Meal plans, recipe charts, portion control diagrams
**Late Pregnancy**	32–36 weeks	Weight management, iron absorption strategies, and late pregnancy care	Practical food tips, anemia charts, and continuity guides

Participants Category based on BMI, Lipid Profile, and Specific Nutrition Education			
**Underweight**	**Normal weight**	**Overweight**	**Dyslipidemia**
-How to gain weight<br>- What food to eat to get the recommended energy as per the RDA<br>- Why is protein essential for you and your baby?<br>- Function of protein<br>-Energy-rich sources of food<br>-Healthy options for fat<br>- Importance of nutrients and their timings<br>-Importance of micronutrients, especially iron, folic acid, calcium, and vitamin D in pregnancy<br>- Why is weight gain essential for you	-How to maintain a healthy weight during pregnancy<br>- Maintaining daily energy intake to reduce the risk of LBW neonates<br>-Methods of improving the bioavailability of food for better absorption of nutrients<br>-Improving nutritional practices to avoid pregnancy risk<br>-Alternate sources of food to meet a particular nutrient demand<br>-The role of protein, iron, folic acid, calcium, and vitamin D in pregnancy	-Good practices of nutrition to maintain a healthy body weight and to have better pregnancy outcomes<br>-The source of food nutrients is dense, over-calorie-dense food<br>-Why you should not try to lose weight in pregnancy<br>-How to meet your calorie and nutrient needs without going overboard<br>-Importance of micronutrients, especially iron, folic acid, calcium, and vitamin D in pregnancy	-Role of weight management in controlling dyslipidemia<br>-The role of omega-3 and their source in improving dyslipidemia<br>-Role and source of fiber and soluble fiber in lowering cholesterol levels<br>-The role of phytonutrients in lowering LDL-c<br>-What are the better sources of fat in dyslipidemia

The nutrition education program was not limited to the counseling sessions above. More details and education were provided on various other aspects, including the benefits of exercise and the ill effects of smoking, alcohol, and drugs in pregnancy.

Supplementary Material (S2)

DATA COLLECTION TOOLS

(APPENDIX A to G)

SCREENING PROFORMA

Name of the Patient:	
UHID:	
Contact No:	
**Patient Details**	**Inclusion Criteria**
Maternal Age in years:	Between 18-40 years
Gestational Age in weeks:	Between 8-14 weeks
Pregnancy: Singleton………. or<br>Multifetal…………	Singleton
Conception: Spontaneous………. or<br>Assisted……….	Spontaneous
Any underlying severe disease:<br>• Diabetes<br>• Hypertension<br>• Tuberculosis<br>• Thyroid<br>• Kidney disease<br>• Heart Disease<br>• Liver Disease<br>• Others:	No Severe Illness

QUESTIONNAIRE

Topic: The Association of Maternal Nutritional Status and Lipid Profile with Perinatal Outcome

Note: Please do share your answer honestly. This will ensure that we make areliable analysis that will help pregnant women in the future.

……………………………………………………………………………………….................

APPENDIX-A

SOCIO-DEMOGRAPHIC ASSESSMENT

1. Name: …………………… **UHID:……………….……… Ph No: ……..…………...**

2. Age: ……yrs. **Height**: ……...ft ……. inches or …………cm or …………m

3. Weight: Pre-Pregnancy**……………kg Current Weight:**…………….. **kg**

4. **Gestational Age** …………….in weeks **Trimester** ………………….

5. **Consanguineous Marriage** ☐ **Non-consanguineous Marriage** ☐

LMP: **USG Dating:**

EDD:

6. What is your religion?

Christianity	Hinduism	Islam	Jainism	Sikhism	Buddhism	Others

7. Food Habits:

Vegetarian	
Non-vegetarian (Egg, Meat, Fish)	
Other	

8. Highest Degree Received:

S. No	Degree	Respondents	Your Spouse
1	Professional/ Post Grad. Or Higher		
2	Graduate		
3	Intermediate or Diploma		
4	High School Certificate		
5	Middle School Certificate		
6	Primary School Certificate		
7	Illiterate		

9. Employment Status:

S.No	Occupation (Job)	Respondents	Your Spouse	Head of the Family
1	Legislators, Senior Officials, Managers			
2	Professionals			
3	Technicians & Associate Professionals			
4	Clerks			
5	Skilled Workers/Shop Sales workers			
6	Skilled Agricultural & Fishery Workers			
7	Craft & Related Trade workers			
8	Plant and Machine Operators/Assembler			
9	Elementary Occupation			
10	Unemployed (Home Maker)			

10. Number of Children you have (please tick the appropriate option)

None so far	
One	
Two	
Three	
Four or more	

11. The number of Family members (Family size):

S.No	Number of Family Members	Tick the appropriate answer
1	1-3 members	
2	4-5 members	
3	6-7 members	
4	7-10 members	
5	10 or more members	

12. Please choose one of the following that best describes your Household Income per annum (per month):

S. No	Household Income (Per month in INR)	Tick the appropriate option
1	Less than 9,226	
2	Between 9,232 to 27,648	
3	Between 27,654 to 46,089	
4	Between 46,095 to 68,961	
5	Between 68,967 to 92,185	
6	Between 92,191 to 184,370	
7	More than 1,84,376	

13. Place of Residence: Rural…………… Urban…………..

14. Address:

APPENDIX-B

ANTHROPOMETRIC MEASUREMENTS

- **Height (in cm):................. (in m): ……………..(in Feet) …………..**
- **Weight (in kg):....................**
- **Body Mass Index (in kg/m^2): ……………………**

APPENDIX- C

24-hour Dietary Intake Questionnaire
Individual Intake Form

Meal	Menu	Ingredients	Amount (g)
Early morning			
Breakfast			
Mid-morning Snacks			
Lunch			
Evening Snacks			
Dinner			
Post Dinner			

APPENDIX-D

FOOD FREQUENCY QUESTIONNAIRE (FFQ)

FOOD GROUPS	Daily	Alternate days	Once a week	Once every 15 days	Once a month	Never
**CEREALS**<br>(Rice, wheat, ragi, bajra, maize, jowar, barley, rice flakes, wheat flour)						
**PULSES AND LEGUMES**<br>Bengal gram, Black gram, green gram, red gram, Lentil (whole as well as dhals), Cowpea, Peas, Rajma, Soybeans, Beans						
**MILK AND MILK PRODUCTS**<br>Milk, Curd, Skimmed milk, Cheese						
**EGG, MEAT & FISH**						
**FRUITS**<br>Mango, Guava, Tomato, Ripe Papaya, Orange. Sweet Lime, Watermelon.						
**GREEN LEAFY VEGETABLES**<br>Amaranth, Spinach, Drumstick leaves, Coriander leaves, Mustard<br>leaves, fenugreek leaves						
**ROOTS & TUBERS**<br>Carrots, Brinjal, Ladies' fingers, Capsicum, Beans, Onion, Drumstick, Cauliflower.						
**NUTS & SEEDS**<br>(Almond, walnut, pistachio)						
**FATS & OILS**<br>Butter, Ghee, Hydrogenated oils,<br>Cooking oils like Groundnut, Mustard, Coconut.						
**SUGAR & JAGGERY**						

APPENDIX-E

KNOWLEDGE-BASED QUESTIONS

(Please tick only ONE option as your answer)

1. When is Nutrition important?

a. Before pregnancy begins
b. During pregnancy
c. After the delivery of the baby
d. All the above
e. Do not Know

2. Food during pregnancy should be

a. Balanced Diet
b. High in Carbohydrate
c. High in Fat
d. High in Protein
e. Do not Know

3. A balanced diet is?

a. A diet with all nutrients such as Energy, Proteins, Fats, Vitamins & Minerals.
b. A diet with lots of fruits and vegetables.
c. A diet that provides sufficient energy.
d. None of the above
e. Do not Know

4. Food requirements during pregnancy are:

a. Same as it was before pregnancy
b. Slightly higher than before the pregnancy
c. Twice as much as before pregnancy
d. Thrice as much as before pregnancy
e. Do not Know

5. What should the food frequency (number of meals) during pregnancy be?

a. 1 meal, 1 snack
b. 1 meal, 2 snacks
c. 2 meals, 2 snacks
d. 3 meals, 2/3 snacks
e. Do not Know

6. What are the good sources of protein-rich foods?

a. Egg, Meat, and Fish
b. Butter
c. Ghee
d. Cereal
e. Do not Know

7. Which nutrients are essential during pregnancy?

a. Calcium
b. Iron and folic acid
c. Protein
d. All the above
e. Both a & b
f. Do not Know

8. How many kilocalories (Kcal) are in 1 gram of sugar (Carbohydrates)?

a. 4 Kcal
b. 6 Kcal
c. 9 Kcal
d. Do not Know

9. Good source of Iron-rich food is?

a. Red Meat / Organ Meat
b. Spinach
c. Both a & b
d. Do not Know

10. Iron absorption in the body is enhanced by?

a. Vitamin A
b. Vitamin C
c. Vitamin D
d. Vitamin E
e. Do not Know

11. Absorption of Iron in the body is inhibited by?

a. Tea
b. Coffee
c. Calcium-rich food
d. All the above
e. Both a & b
f. Do not Know

12. A good source of Vitamin C is

a. Lemon
b. Banana
c. Apple
d. All the above
e. Do not Know

13. Iron is better absorbed from

a. Animal sources (Heme Iron)
b. Plant sources (Non Heme Iron)
c. Both a and b
d. None of the above
e. Do not Know

14. Calcium is necessary during pregnancy for

a. Good bone health of the mother
b. Calcification of baby teeth and bones
c. Both a and b
d. None of Above
e. Do not Know

15. Which are the food sources of Vitamin D?

a. Fruit
b. Vegetables
c. Egg yolk/ Fish Liver/ Fortified Cereals
d. None of the above
e. Do not Know

16. What do you understand by RDA in terms of Nutrition?

a. Record Daily Amount
b. Recorded Daily Allowance
c. Recommended Dietary Allowance
d. Do not Know

17. Which of the following is the best source of Folic Acid?

a. Leafy green vegetables
b. Eggs
c. Legumes
d. All the above
e. Do not know

18. Nutrient deficiency during pregnancy could affect

a. Health status of mothers
b. Health status of fetus (baby)
c. Both a and b
d. None of the above
e. Do not Know

19. A deficiency of iron during pregnancy can lead to

a. Anemia
b. Low Birthweight Babies
c. Pre-term delivery
d. All the above
e. Do not Know

20. Neural Tube Defect is caused by:

a. Deficiency of Iron
b. Deficiency of Vitamin-A
c. Deficiency of Folic Acid
d. Deficiency of Vitamin-B
e. Do not Know

……………………………………………………………………………………………

APPENDIX-F

PRACTICE-BASED QUESTIONS

(Please tick only one option as your answer)

1. Do you consult your Obstetrician/Gyenocologist for nutritional guidance?
a) Yes b) No

2. Is your current food intake (meal) slightly higher than before pregnancy?
a. Yes b) No

3. Do all your meals contain at least one fruit and/or vegetable?
a) Yes b) No

4. Do you prefer packed food over home-cooked food?
a) Yes b) No

5. Do you skip your meal (Breakfast or Lunch or Dinner)?
a) Yes b) No

6. Do you pay attention to what you eat?
a) Yes b) No

7. Do you control the portion size of your meal?
a) Yes b) No

8. Do you take milk/ milk products to meet your calcium requirements?
a) Yes b) No

9. **Do you get your weight and/ or BMI assessed at every antenatal visit?**
a) Yes b) No

10. Do you smoke during your pregnancy?
a) Yes b) No

11. **Do you consume alcohol during your pregnancy?**

a) Yes b) No

12. Have you received any nutritional counseling before?

a) Yes b) No

13. Do you take three meals and 2/3 snacks daily as your meal pattern?

a) Yes b) No

14. Do you eat an additional amount of protein during pregnancy than before?

a) Yes b) No

15. Do you take any of the following supplements?

a) Yes b) No

- Vitamin D
- Calcium
- Iron-Folic acid

....

Thank you for your co-operation

APPENDIX-G

LIPID PROFILE & VITAMIN D

Component	1st Reading (8-14 weeks)	Follow-up reading (32-36 weeks)
Cholesterol		
Triglycerides		
LDLc		
HDLc		
Cholesterol/HDL Ratio		
Vitamin D		

GROWTH SCAN at 32-36 WEEKS

Measure of	Readings
Abdominal circumference (AC),	
Head circumference (HC),	
Femur length (FL)	
Biparietal diameter (BPD)	
Estimated Fetal Weight	

PERINATAL OUTCOME DETAILS

Component		
APGAR Score (1min & 5 min)		
Birth Weight of Neonate		
Sex of the Child		
Mode of Delivery		
Date of Delivery		
Gestational Age at Birth		

OTHER DETAILS FROM ANC CARD OR REPORT (IF AVAILABLE)

Component	1st Reading (8-14 weeks)	Follow-up reading (32-36 weeks)
Blood Pressure		
Random Blood Sugar		
OGTT		
HbA1c		
Hemoglobin		
Double Marker (B-Hcg)		
PAPP-A		
Quadruple Marker		
TSH		
T3		
T4		
NT/NB		

Supplementary Table 1

ST 1: Baseline characteristics of participants in the intervention and control groups were comparable with no significant differences (p>0.05). Data are presented as mean ± standard deviation for continuous variables and percentages for categorical variables.

**Characteristic**	**Intervention group (n=88)**	**Control group (n=85)**
Age (years)	27.23 ± 4.25	27.59 ± 4.23
Height (cm)	154.89 ± 5.01	154.61 ± 6.48
Pre-pregnancy weight (kg)	56.78 ± 11.89	54.07 ± 9.42
Pre-pregnancy BMI (kg/m²)	23.57 ± 4.37	22.61 ± 3.57
Gestational age at baseline (Median)	12 weeks 3 days	12 weeks 4 days
Gravidity ((n) & %)<br>*Primigravida*<br>*Multigravida*	 (43) 48.86%<br>(45) 51.14%	 (44) 51.76%<br>(41) 48.23%
Religion (%)<br>- *Hinduism*<br>- *Islam*<br>- *Christian*<br>- *Sikh*	 56.7%<br>43.3%<br>-<br>-	 56.5%<br>41.1%<br>1.2%<br>1.2%
Food Habit (%)<br>- *Vegetarian*<br>- *Non-Vegetarian*	 33%<br>67%	 36.5%<br>63.5%
Educational Qualification (*n*)<br>- *Post Graduation*<br>- *Graduation*<br>- *Intermediate/ Diploma*<br>- *High School*<br>- *Middle School*<br>- *Primary School*<br>- *Illiterate*	 12<br>26<br>21<br>19<br>09<br>00<br>01	 09<br>37<br>30<br>05<br>02<br>01<br>01
Socio-economic Status (SES) (*n*)<br>- *Upper-middle class (SES II)*<br>- *Lower-middle class (SES III)*	 9<br>79	 10<br>75
Blood Pressure (*mean* ± *S.D*)<br>- Systolic Blood Pressure (mm/Hg)<br>- Diastolic Blood Pressure (mm/Hg)	 108 ± 12<br>72 ± 8	 108 ± 8<br>72 ± 6

***[Note:** We do not want to publish this table with this manuscript, as this is being used in another manuscript under review (part of the Ph.D. Thesis work of the first author)]*
